# 

**Published:** 1872-08-15

**Authors:** 


Money Receipts to Sept. 1st, 1872.—Dr. Wm. Low, $3.00; Dr. N. Senn, $3.00; Dr. L. Asire, $3.00; Dr. J. M. Steele, $5.00; Dr. E. W. Edwards, $3.00; Dr. E. S. Cleveland, $6.00; Dr. C. Marsh, $15.00; Dr. M. Parker, $3.00; Dr. W. Blanchard, $3.00; Dr. J. W. Fink, $3.00; Dr. Orin Peck, $6.00; Dr. A. Gaslin, $6.00; Dr. W. H. Searles, $3.00; Dr. G. W. Lawrence, $5.00; Dr. E. F. Greenleaf, $5.00; Dr. D. Lichty, $4.00; Dr. F. R. Payne, $3.00.

